# Complete Genome Sequence of the Industrial Fast-Acidifying Strain Streptococcus thermophilus N4L

**DOI:** 10.1128/MRA.01029-18

**Published:** 2018-08-30

**Authors:** Lucas Proust, Valentin Loux, Véronique Martin, Cristian Magnabosco, Martin Pedersen, Véronique Monnet, Vincent Juillard

**Affiliations:** a Micalis Institute, INRA, AgroParisTech, Université Paris-Saclay, Jouy-en-Josas, France; b MaIAGE, INRA, Université Paris-Saclay, Jouy-en-Josas, France; c Procelys, Lesaffre Group, Maisons-Alfort, France; d Sacco S.r.l., Cadorago, Italy; University of Maryland School of Medicine

## Abstract

Streptococcus thermophilus is one of the most used dairy starters for the production of yogurt and cheese. We report here the complete genome sequence of the industrial strain S. thermophilus N4L, which is used in dairy technology for its fast-acidifying phenotype.

## ANNOUNCEMENT

Streptococcus thermophilus is a well-known lactic acid bacterium (LAB) widely used for the production of dairy products in association with other LAB species, such as Lactococcus lactis and Lactobacillus delbrueckii subsp. bulgaricus, for cheese and yogurt production, respectively. It belongs to the salivarius group and recently evolved from a commensal ancestor (1). Nowadays, it can be naturally found all around the world in dairy production facilities, whether artisanal or industrial (2). For industrial use, strains are carefully selected for their ability to ferment milk and confer desirable properties to the final product. In particular, due to the high prevalence of the cell wall proteinase *prtS* gene (1) in industrial isolates of S. thermophilus, they are often associated with higher productivity in milk when compared to their wild or traditional counterparts. The proteinase PrtS is responsible for the breakdown of caseins into peptides, which are used as sources of amino acids, thus allowing faster growth and acidification than do PrtS-negative strains. These released peptides are then internalized inside the bacterial cell primarily by the Ami oligopeptide transport system, which includes one or several peptide-binding proteins depending on the strain (3).

S. thermophilus N4L is an industrial strain used precisely for its fast-acidifying phenotype. A stationary-phase culture (M17 with 2% lactose) was subjected to genomic DNA (gDNA) extraction using the GenElute bacterial gDNA kit (Sigma-Aldrich, USA) following the protocol indicated by the manufacturer. Its gDNA was then sequenced (Macrogen, Inc., South Korea) on a PacBio RS II system (Pacific Biosciences, USA) using single-molecule real-time (SMRT) sequencing technology (4). A total of 150,292 polymerase reads were obtained, displaying an average length of 11,243 bp. These reads, after quality filtering and adapter trimming, were converted into 213,700 subreads with an average length of 7,626 bp. They were subsequently used for *de novo* genome assembly. For that purpose, Canu version 1.6 was employed with all default settings for subread correction, trimming, and assembly (5). The resulting genome corresponded to a unique circular chromosome of 1,831,756 bp with an 889× coverage depth and a G+C content of 39.05%.

Annotation was performed with the AGMIAL annotation platform (6). It revealed the presence of 2,300 open reading frames (ORFs), including 67 tRNAs and 18 rRNAs. This sequencing work also confirmed the presence of the genomic island encoding the *prtS* gene, as well as two copies of the oligopeptide-binding proteins AmiA1 and AmiA3, which explains the high acidification rates in milk observed with this strain. All of the genes required for natural competence are also present in the genome (7, 8), suggesting that the strain could theoretically be naturally transformable.

### Data availability.

The complete genome sequence of S. thermophilus strain N4L has been deposited in GenBank under the accession no. LS974444 (BioProject no. PRJEB27286).
